# COVID-19 pandemic and initiation of treatment for atrial fibrillation: a nationwide analysis of claims data

**DOI:** 10.1186/s12872-023-03614-z

**Published:** 2023-12-08

**Authors:** Lanting Yang, Shangbin Tang, Meiqi He, Jingchuan Guo, Nico Gabriel, Gretchen Swabe, Walid F Gellad, Utibe R Essien, Samir Saba, Emelia J Benjamin, Jared W Magnani, Inmaculada Hernandez

**Affiliations:** 1 Department of Pharmacy and Therapeutics, University of Pittsburgh School of Pharmacy, Pittsburgh, PA USA; 2 Division of Clinical Pharmacy, Skaggs School of Pharmacy and Pharmaceutical Sciences, University of California, San Diego, La Jolla, CA USA; 3 Department of Pharmaceutical Outcomes and Policy, University of Florida College of Pharmacy, Gainesville, FL USA; 4 Division of Cardiology, Department of Medicine, University of Pittsburgh School of Medicine, Pittsburgh, PA USA; 5 Division of General Internal Medicine, Center for Health Equity Research and Promotion, VA Pittsburgh Healthcare System Pittsburgh, University of Pittsburgh School of Medicine, Pittsburgh, PA USA; 6 Division of General Internal Medicine and Health Services Research, David Geffen School of Medicine, Center for the Study of Healthcare Innovation, Implementation & Policy, University of California, Greater Los Angeles VA Healthcare System, Los Angeles, Los Angeles, CA USA; 7 Department of Epidemiology, Boston University School of Public Health, Department of Medicine, Boston Medical Center and Boston University Chobanian and Avedisian School of Medicine, Boston University, Boston, MA USA

**Keywords:** Atrial fibrillation, Covid-19 pandemic, Treatment initiation, Oral anticoagulation, Electrical cardioversion

## Abstract

**Background:**

The COVID-19 pandemic profoundly disrupted the delivery of medical care. It remains unclear whether individuals diagnosed with new onset disease during the pandemic were less likely to initiate treatments after diagnosis. We sought to evaluate changes in the treatment initiation of patients newly diagnosed with atrial fibrillation (AF) after the onset of the COVID-19 pandemic.

**Methods:**

In this retrospective cohort study, we identified individuals with incident AF from 01/01/2016–09/30/2021 using Optum’s de-identified Clinformatics® Data Mart Database. The primary outcome was initiation of oral anticoagulation (OAC) within 30 days of AF diagnosis. Secondary outcomes included initiation of OAC within 180 days of diagnosis, initiation of warfarin, direct oral anticoagulants (DOACs), rhythm control medications and electrical cardioversion within 30 days of diagnosis. We constructed interrupted time series analyses to examine changes in the outcomes following the onset of the pandemic.

**Results:**

A total of 573,524 patients (age 73.0 ± 10.9 years) were included in the study. There were no significant changes in the initiation of OAC, DOAC, and rhythm control medications associated with the onset of the pandemic. There was a significant decrease in initiation of electrical cardioversion associated with the onset of the pandemic. The rate of electronic cardioversion within 30 days of diagnosis decreased by 4.9% per 1,000 patients after the onset of the pandemic and decreased by about 35% in April 2020, compared to April 2019, from 5.53% to 3.58%.

**Conclusion:**

The COVID-19 pandemic did not affect the OAC initiation within 30 days of AF diagnosis but was associated with a decline in the provision of procedures for patients newly diagnosed with AF.

**Supplementary Information:**

The online version contains supplementary material available at 10.1186/s12872-023-03614-z.

## Introduction

The COVID-19 pandemic had a profound impact on health care access. A robust body of literature has documented declines in patient encounters with the health care system in the early days of the COVID-19 pandemic, which resulted in apparent decreases in rates of clinical events, even for life-threatening conditions [1–5]. Decreased use of imaging tests, laboratory services or provider-administered drugs have also been described in the literature [6–8]. Although this emerging literature is crucial to understand the negative impacts of the COVID-19 pandemic beyond COVID cases and deaths, prior studies mostly focused on the evaluation of clinical services that required access to health care facilities, rather than evaluating disruptions in outpatient pharmacotherapy. Additionally, few studies have evaluated patterns of health services delivered to patients with new onset disease during the COVID-19 pandemic. As a result, it remains unclear to what extent pandemic disruptions of health care access have resulted in forgone or delayed initiation of treatment for new onset disease.

Our study used US nationwide data from Optum’s de-identified Clinformatics® Data Mart Database to evaluate changes in treatment initiation in patients newly diagnosed with atrial fibrillation (AF). AF is a crucial disease to evaluate disruptions in care associated with the COVID-19 pandemic because early initiation of oral anticoagulation and consistent adherence is essential to prevent ischemic stroke events [9, 10]. Additionally, the coexistence of COVID-19 and AF creates a potentially deadly combination, substantially increasing the risk of pulmonary embolism, stroke, and venous thromboembolism [11–13]. The adverse outcomes observed when these two conditions converge underscore the significance of promptly initiating treatments for patients with AF.

## MethodS

### Data sources and study population

**Fig. 1:**
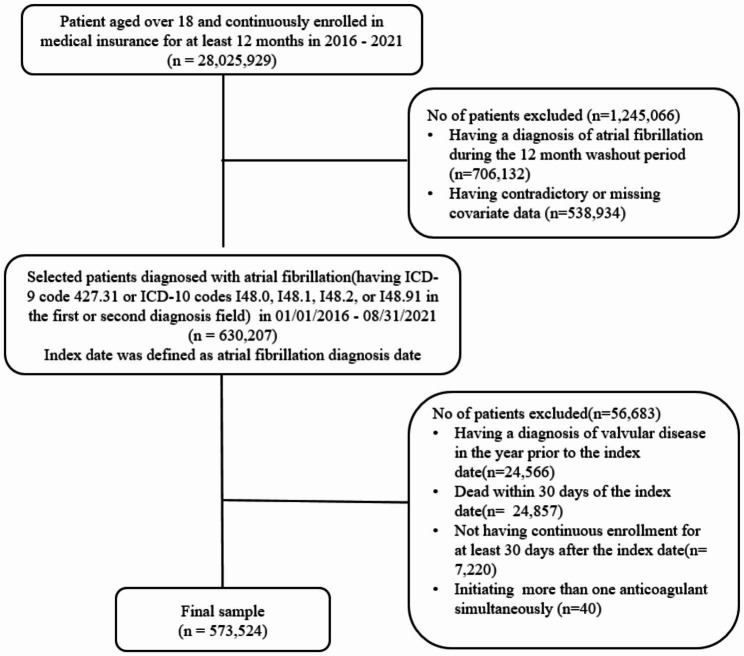
Overview of the Sample Selection. Atrial fibrillation was defined as having an inpatient or outpatient claim with International Classification of Diseases Ninth Revision (ICD-9) code 427.31 or International Classification of Diseases Tenth Revision (ICD-10) codes I48.0, I48.1, I48.2, or I48.91 in the first or second diagnosis field. Valvular disease was defined as having ICD-9 codes 394.0, V43.3 or ICD-10 codes I05.0, Z95.2 at any diagnosis field

We obtained claims data between 01/01/2016–09/30/2021 from Optum’s de-identified Clinformatics® Data Mart Database (most recent data available at the time of analysis). Optum’s de-identified Clinformatics® Data Mart Database data are derived from administrative health claims for large commercial and Medicare Advantage health plans. The data included verified, adjudicated, and de-identified medical and pharmacy claims for a geographically diverse population spanning all 50 states. We selected the study population in six steps (Fig. 1). First, we selected patients aged over 18 years and who were continuously enrolled for at least 12 months in 1/1/2016-8/31/2021 (n = 28,025,929). Second, we excluded patients who had a diagnosis of AF in the first 12 months of continuous enrollment or who had incomplete covariate data (n = 1,245,066). This ensured we had 12 months of complete data prior to AF diagnosis for the definition of baseline characteristics and the exclusion of prevalent AF patients. AF was defined as having an inpatient or outpatient claim with International Classification of Diseases Ninth Revision (ICD-9) code 427.31 or International Classification of Diseases Tenth Revision (ICD-10) codes I48.0, I48.1, I48.2, or I48.91 in the first or second diagnosis fields [14]. Third, we selected patients who were newly diagnosed with AF after the 12-month washout period (n = 630,207). The index date was defined as the AF diagnosis date. Fourth, we excluded patients who had a diagnosis of valvular disease in the 12 months prior to the index date (n = 24,566). Valvular disease was defined as having ICD-9 codes 394.0, V43.3 or ICD-10 codes I05.0, Z95.2 in any diagnosis field [15]. Fifth, we excluded patients who died (n = 24,857) or did not have continuous enrollment (n = 7,220) for at least 30 days after the index date. This ensured we did not have missing data for the primary outcome. Finally, we excluded patients who initiated multiple anticoagulants on the same day (n = 40). The final sample included 573,524 eligible patients with incident AF between 01/01/2016–09/30/2021. Patients were followed from the index date for 180 days or until death, disenrollment, initiation of outcomes, or end of the study (9/30/2021). The Institutional Review Board at the University of California, San Diego approved this study as exempt as de-identified data were used in analyses.

### Outcomes

The primary outcome of interest was oral anticoagulation initiation within 30 days of AF diagnosis. This was defined as filling a prescription for warfarin or a direct oral anticoagulant (DOAC) within 30 days of the AF diagnosis. Secondary outcomes were initiation of oral anticoagulation within 180 days of AF diagnosis, initiation of warfarin, initiation of DOACs, and other AF treatment measures, including initiation of rhythm control medications and electrical cardioversion, all within 30 days of AF diagnosis. The definition of oral anticoagulant initiation within 180 days of AF diagnosis enabled for the capture of treatment initiation among individuals who may have been hospitalized at the time of diagnosis or who may have received free samples from providers, whose early initiation during the inpatient admission or through free samples would not be captured in Medicare pharmacy claims. This expanded time frame also enabled capture of treatment initiation among individuals who may have had to overcome administrative barriers for insurance coverage, such as prior authorization. Electrical cardioversion was defined as having a claim with CPT-4 code 92,960 or 92,961. Initiation of rhythm control medications was defined as filling a prescription for amiodarone, disopyramide, dofetilide, dronedarone, flecainide, mexiletine, propafenone, quinidine, and sotalol. All outcomes were reported in 30-day-intervals.

### Independent variables

The main independent variable of interest was time after the World Health Organization declaration of the pandemic (3/11/2020) [16]. Covariates included patient demographics, setting of AF diagnosis, clinical characteristics and health insurance factors identified as of index date. Demographic characteristics included age, gender, race, and ethnicity. Age was categorized into ≥ 75 and ˂75 years subgroups. Race and ethnicity were categorized into non-Hispanic White, non-Hispanic Black, Hispanic, and other. Race and ethnicity data were collected using public records and imputation with commercial algorithms developed using census data with first and last names [17]. Setting of AF diagnosis included inpatient and outpatient and was defined using the place of service code in the claim of AF diagnosis. Diagnoses claims with place of service code 21 were categorized as inpatient and claims that did not originate from the inpatient setting were categorized as outpatient.

Clinical characteristics included CHAD2DS2-VACs score and HAS-BLED score. CHA2DS2-VASc predicts stroke risk in AF patients and gives one point for each of the following factors: age 65 to 74 years, congestive heart failure, hypertension, diabetes mellitus, vascular disease, and female sex. Two points are given for age ≥ 75 years and a history of stroke or transient ischemic attack [18]. HAS-BLED scores estimate the risk of major bleeding with anticoagulation and are calculated on the basis of following risk factors: age > 65 years, hypertension, renal disease, liver disease, stroke and bleeding history, drugs or alcohol use [19]. Since claims data does not include international normalized ratio (INR) information, we calculated HAS-BLED scores as the sum of all above factors except for labile INR, as previously done in the literature [20, 21]. Health insurance factors included Medicare enrollment, dual eligibility, receipt of low-income subsidy, enrollment in a health savings account, and type of health plan including health maintenance organization, preferred provider organization, point of service, exclusive provider organization, and other.

### Statistical analysis

We described patient characteristics in the overall cohort. For each 30-day-interval, we reported the incidence rate of each outcome calculated as the proportion of patients at risk in each 30-day interval who experienced the outcome in the given interval. For the primary outcome of oral anticoagulation initiation, we performed subgroup analyses by age, gender, race, ethnicity, and clinical setting of AF diagnosis.

We performed interrupted time series analyses to formally test changes in outcomes following the onset of the COVID-19 pandemic. Interrupted time series analysis is the preferred methodology to evaluate the effects of public health interventions or disruptions introduced at a population level within a well-defined timeframe, as it is the case of the COVID-19 pandemic [22–24]. In our interrupted time series model, the outcome variable was regressed against a continuous variable for time (measured in 30-day-intervals), an indicator variable for time after the onset of the COVID-19 pandemic, and the interaction variable between the two of them. The indicator variable for time after the onset of the COVID-19 pandemic represents whether there is a change in the level of the outcome, that is, a change immediately after the breakpoint (represented by a change in the intercept of the regression model). The interaction variable between continuous time and time after the onset of the COVID-19 pandemic represents changes in the trend of the outcome. In essence, it reflects alterations in the slope of the regression model, enabling us to identify prolonged changes in the outcome over time. The model is not adjusted for other covariates. Two-tailed P-values less than 0.05 were defined as statistically significant. Analyses were performed using SAS version 9.4 (SAS Institute Inc., Cary, NC).

## ResultS

### Study sample

The final cohort included 573,524 patients newly diagnosed with AF. The mean (SD) age for the cohort was 73 ± 10.9 years, 48.0% of study participants were female and 74.2% were Non-Hispanic White (Table 1). Medicare beneficiaries accounted for 84.5% of the study participants.

**Table 1:** Baseline Patient Characteristics

Variable	Overall Cohort (n = 573,524)
**Demographics**	
Female, No. (%)	275,576 (48.0)
Age, Mean ± Std.	73.0 ± 10.9
Age, years	
< 50, No. (%)	20,446 (3.6)
50–64, No. (%)	76,457 (13.3)
65–74, No. (%)	197,686 (34.5)
>=75, No. (%)	278,935 (48.6)
Race and Ethnicity^a^	
Non-Hispanic White, No. (%)	425,699 (74.2)
Non-Hispanic Black, No. (%)	57,674 (10.1)
Hispanic, No. (%)	49,474 (8.6)
Other, No. (%)	40,677 (7.1)
**Clinical Characteristics**	
CHA_2_DS_2_-VASc Score^b^	
Low Risk, No. (%)	28,789 (5.0)
Moderate Risk, No. (%)	54,786 (9.6)
High Risk, No. (%)	489,949 (85.4)
HAS-BLED Score^c^	
0, No. (%)	24,286 (4.2)
1–2, No. (%)	273,960 (47.8)
>=3, No. (%)	275,278 (48.0)
**Health Insurance Factors**	
Medicare, No. (%)	484,852 (84.5)
Medicare/Medicaid Dual Eligible, No. (%)	33,996 (5.9)
Receipt of Low-Income Subsidy, No. (%)	45,690 (8.0)
Health Savings Account, No. (%)	15,416 (2.7)
Plan Type	
Health Maintenance Organization, No. (%)	146,010 (25.5)
Preferred Provider Organization, No. (%)	43,365 (7.6)
Point of Service, No. (%)	59,600 (10.4)
Exclusive Provider Organization, No. (%)	8673 (1.5)
Other, No. (%)	315,876 (55.1)

Abbreviations: AF, atrial fibrillation

a Reported race and ethnicity, dependent on statistical de-identification rules for race based on geography, Other race included Asian and Unknown

b CHA2DS2-VASc gives one point for each of the following factors: age 65 to 74 years, congestive heart failure, hypertension, diabetes mellitus, vascular disease, and female sex, and two points for age ≥ 75 years, and a history of stroke or transient ischemic attack. Female with CHA_2_DS_2_-VASc Score < 2, =2 and > 2 were defined as low risk, moderate risk and high risk, respectively. Male with CHA_2_DS_2_-VASc Score < 1, =1 and > 1 were defined as low risk, moderate risk and high risk, respectively

c HAS-BLED is calculated on the basis of following risk factors: age > 65 years, hypertension, renal disease, liver disease, stroke and bleeding history, drugs or alcohol use. Because claims data do not include international normalized ratio (INR) information, we calculated HAS-BLED score as the sum of all above factors except for labile INR.

### Changes in initiation of oral anticoagulation

**Fig. 2:**
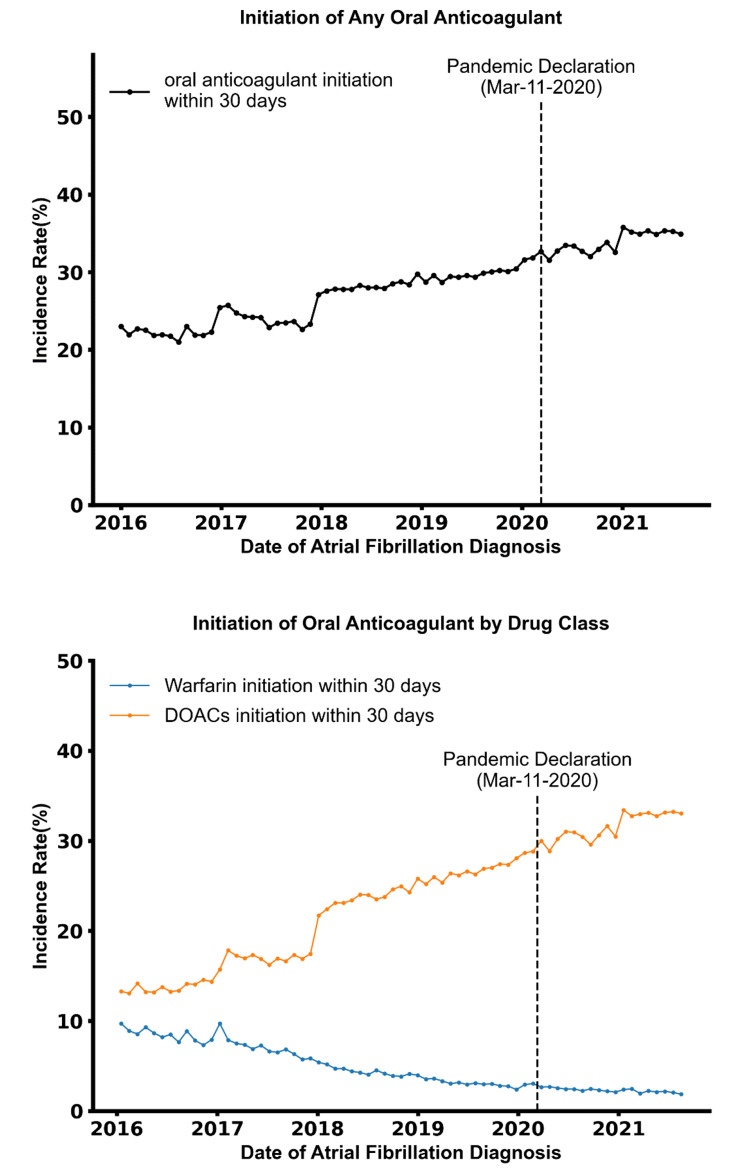
Initiation of Any Oral Anticoagulants within 30 Days of Atrial Fibrillation Diagnosis. Abbreviations: DOACs, direct oral anticoagulants. The upper panel shows trends in the initiation any oral anticoagulant agent within 30 and 180 days of atrial fibrillation diagnosis. The lower panel shows trends in the initiation of warfarin and direct oral anticoagulants separately. Data are shown in 30-day intervals, from 01/01/2016 to 09/30/2021.

The proportion of patients initiating oral anticoagulation within 30 days of diagnosis increased from an average of 22.1% in 2016 to 34.9% in 2021 (Fig. 2, upper panel). The proportion of patients initiating oral anticoagulation within 180 days of diagnosis increased from 35.5% to 2016 to 48.5% in 2021. There were no significant changes in the initiation of oral anticoagulation within 30 days of AF diagnosis following with the onset of the pandemic (p-value for level change > 0.05 and p-value for trend change > 0.05, Table 2). The trends in the initiation of oral anticoagulation were consistent between patients diagnosed with AF in the inpatient and outpatient setting (Supplemental Fig. 1) and across subgroups defined by age, gender, race and ethnicity (Supplemental Fig. 2). The trends of changes in the proportion of patients initiating oral anticoagulation within 30 days and 180 days of AF diagnosis are similar in both the inpatient and outpatient settings (Supplemental Fig. 1). Specifically, the proportion of patients initiating oral anticoagulation within 30 days (and 180 days) of diagnosis increased from 22.7% (36.1%) in 2016 to 37.2% (49.9%) in 2021 for inpatient setting and from 21.9% (35.2%) to 33.8% (47.8%) in the outpatient setting.

**Table 2:** Results of Interrupted Time Series Analysis

Initiation of outcomes within 30 days of AF diagnosis	Level Change(95%CI), per 1000 patients^a^	P-Value	Trend Change(95% CI), per 1000 patients ^b^	P-Value	
**Primary Outcome**					
Oral anticoagulation	3.2(-8.5,14.9)	0.59	1.0(-0.9,1.1)	0.85	
**Secondary Outcomes**					
Warfarin	7.6(2.2,12.9)	< 0.01	1.1(0.6,1.6)	< 0.01	
DOAC	-4.4(-16.7,8.0)	0.49	-1.0(-2.1,0.1)	0.07	
Rhythm control medications	-1.1(-2.8,0.6)	0.20	0.1(0.0,0.3)	0.14	
Electrical cardioversion	-4.9(-9.3,-0.6)	0.02	0.1(-0.2,0.5)	0.45	

Abbreviations: DOAC, direct oral anticoagulant

a The level change represents the abrupt change in the outcome immediately after the onset of the COVID-19 pandemic and is given by the indicator variable for the period after 3/11/2020

b The trend change represents the change in the slope of the outcome after the onset of the COVID-19 pandemic and is given by the interaction between the continuous variable for time and the indicator variable for the period after 3/11/2020

### Changes in initiation of DOAC, warfarin, rhythm control medications and electrical cardioversion

The proportion of patients initiating DOAC within 30 days of diagnosis increased from an average of 13.7% in 2016 to 32.7% in 2021 (Fig. 2, lower panel). The proportion of patients initiating warfarin within 30 days of diagnosis decreased from an average of 8.4% in 2016 to 2.2% in 2021. There were no significant changes in DOAC initiation after the onset of the pandemic (p-value for level change > 0.05 and p-value for trend change > 0.05, Table 2). There was, however, an increase in the trend of warfarin initiation following the pandemic onset (p value < 0.01).

**Fig. 3:**
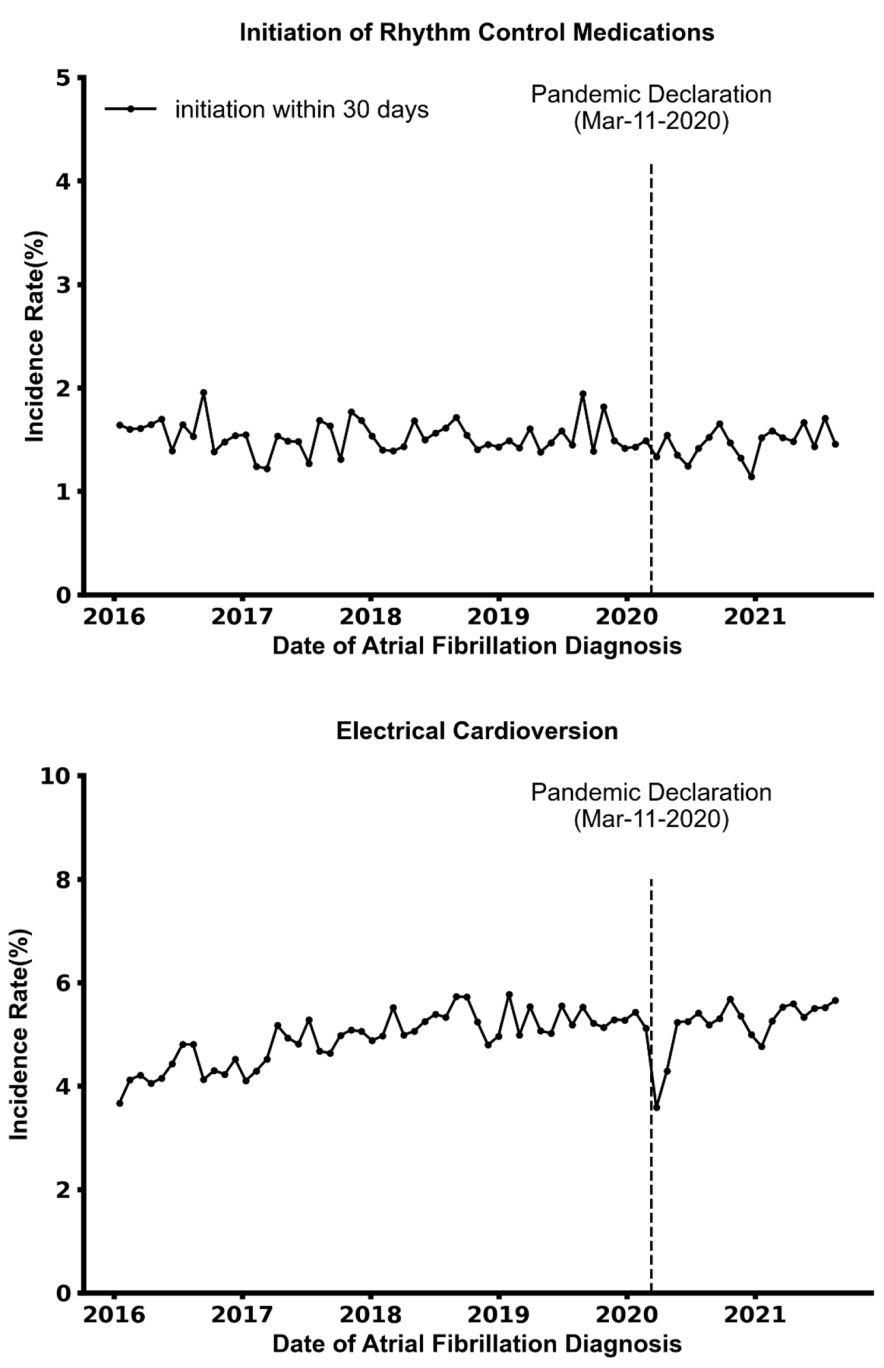
Initiation of Rhythm Control Medications, Electrical Cardioversion within 30 Days of Atrial Fibrillation Diagnosis. The upper panel shows trends in the initiation of rhythm control therapy within 30 days and 180 days of atrial fibrillation diagnosis. The lower panel shows trends in electrical cardioversion procedures within 30 days of atrial fibrillation diagnosis. All outcomes are expressed in 30-day intervals, from 01/01/2016 to 09/30/2021

The rates for initiation of rhythm control medications were similar across the study period (Fig. 3, upper panel). The proportion of patients newly diagnosed with AF who underwent electrical cardioversion increased from an average of 4.3% in 2016 to 5.3% in 2021 (Fig. 3, lower panel). There was a statistically significant decrease in the level of electrical cardioversion immediately after the onset of the pandemic (p value = 0.02, Table 2). Specifically, the rate of electronic cardioversion within 30 days of AF diagnosis decreased by 4.9% (95% CI 0.6%-9.3%) per 1000 patients after the onset of the pandemic. The rate of electronic cardioversion within 30 days of AF diagnosis decreased by about 35% in April, 2020, compared to April 2019, from 5.53% to 3.58%.

## Discussion

In this retrospective analysis, we observed no changes in the initiation of medications for patients newly diagnosed with AF following the onset of the pandemic, including anticoagulation and antiarrhythmic therapies. The findings were consistent across age, gender, race, and ethnicity subgroups. However, we observed an immediate decrease in the level of electronic cardioversion procedures after the onset of the COVID-19 pandemic.

Several studies have reported the COVID-19 pandemic led to delayed or forgone care in patients with chronic diseases [25–33]. For example, one recent study based on French data observed a significant decrease in the initiation of antihypertensive therapy [27]. Prior US-based studies have also reported significant decreases in initiation of treatments in patients with end-stage renal diseases and liver diseases during the pandemic [25, 28]. The therapies evaluated in previous studies, however, are provider-administered, as opposed to outpatient pharmacotherapies obtained in community pharmacies assessed in our analysis. The decreased provision of provider-administered therapies in the early days of the COVID-19 pandemic may be explained by to fear of contagion or overburdened health systems, which would affect the dispensing of outpatient drugs to a lesser extent.

Our study found an amelioration in the rates of warfarin initiation following the onset of the pandemic. However, this finding may not be clinically significant and may not be associated with the COVID-19 pandemic [34]. It is possible the downward trend of warfarin initiation over time may have slowed down as the proportion of patients initiating warfarin approached zero. In fact, a visual inspection of the data suggests the rates of warfarin initiation may soon plateau. Initiation rates may not decrease below a minimum proportion of patients who represent individuals who may begin warfarin due to potential contraindications for DOAC treatment.

Our analysis found a decrease in the rates of electrical cardioversion immediately after the onset of the COVID-19 pandemic. This observed decrease is consistent with previous studies that found decreases in surgical procedures after March 2020 [32]. Several factors may explain why the COVID-19 pandemic disrupted the delivery of procedures but not of outpatient pharmacological treatments. These reasons include provider-initiated cancellations of appointments as health systems were overwhelmed with COVID-19 cases, patient-initiated cancellations for fear of exposure to the virus in healthcare settings, or increased emphasis on the delivery of medical care through telemedicine [35]. Our findings suggest the rate of electrical cardioversion returned to pre-pandemic levels in 2021. Future studies should evaluate if the patients who would have undergone electrical cardioversion under normal circumstances, eventually received the procedure.

Our study has important implications beyond the findings of the disruptions of care associated with the COVID-19 pandemic. Our results shed light on temporal trends in the initiation of therapy for AF and demonstrate that while the proportion of individuals newly diagnosed with AF who receive oral anticoagulation has increased, AF remains largely undertreated. This finding is consistent with previous literature that also used Optum’s de-identified Clinformatics® Data Mart Database data to demonstrate less than 40% of individuals with AF initiated oral anticoagulation within 6 months of diagnosis [36].

Our study is subject to some limitations. First, claims data did not capture prescriptions not covered by insurance, such as warfarin obtained through $4 generic programs [37, 38]. This could have resulted in an underestimation of the treatment initiation rate but should not impact temporal trends in treatment initiation or changes after the onset of the pandemic. Second, the claims data had limited information about patient sociodemographic status and these factors have been shown to influence treatment initiation [39]. Therefore, it is possible the absence of this information may have led to residual confounding in our results. Third, there are limitations and the potential for misclassification by using ICD codes to identify patients with incident AF, which could possibly impact the accuracy of our results. Fourth, our data are limited to individuals with commercial insurance and Medicare Advantage and thus, our findings may not generalize to patients without commercial insurance or the approximately 55% of Medicare beneficiaries enrolled in Medicare fee-for-service [40].

## Conclusion

The initiation of oral anticoagulation within 30 days of AF diagnosis was not disrupted by the onset of the pandemic. However, there was a significant decline in the proportion of patients who underwent electrical cardioversion in the early months of the COVID-19 pandemic. Our study adds important evidence on the impact of the COVID-19 pandemic on the delivery of health care and demonstrates the initiation of pharmacotherapy was not as disrupted as the delivery of provider services.

### Electronic supplementary material

Below is the link to the electronic supplementary material.

Supplementary Material 1

## Data Availability

The datasets generated and/or analyzed during the current study are available from the corresponding author upon reasonable request.

## Abbreviations

AF: Atrial fibrillation
OAC: Oral anticoagulation
DOAC: Direct oral anticoagulant
ICD-9: International Classification of Diseases Ninth Revision
ICD-10: International Classification of Diseases Tenth Revision
SD: Standard deviation
