# Supplementary material for: COVID-19 pandemic and initiation of treatment for atrial fibrillation: a nationwide analysis of claims data
Source: BMC Cardiovasc Disord. 2023 Dec 8;23:604. doi: 10.1186/s12872-023-03614-z (PMC10704685; doi:10.1186/s12872-023-03614-z)
Supplement: Supplementary file 1 — Supplementary Material 1 [file 12872_2023_3614_MOESM1_ESM.docx]

**Supplemental Material**

**Table of Contents**

Supplemental Figure 1. Initiation of Oral Anticoagulation, by Setting of Diagnosis.

Supplemental Figure 2. Initiation of Oral Anticoagulation by Age and Race and Ethnicity.

Supplemental Figure 1. Initiation of Oral Anticoagulation, by Setting of Diagnosis.


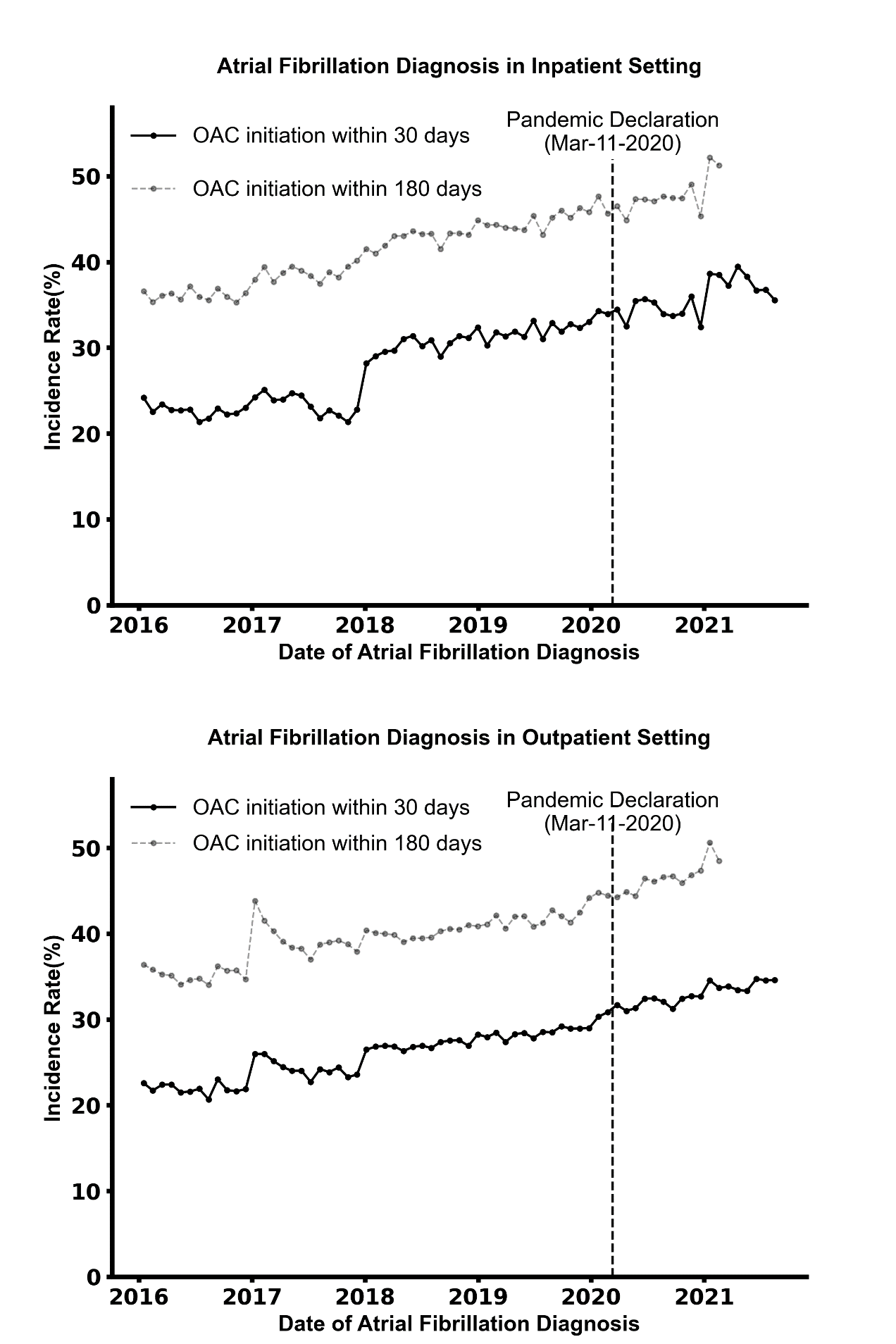


Abbreviations: OAC, oral anticoagulation.

The upper panel shows the trends in the incidence rates of initiation of oral anticoagulants within 30 and 180 days or fibrillation diagnosis. The upper panel represents data for patients diagnosed with AF in the inpatient setting, the lower panel represents data for patients diagnosed in the outpatient setting. Data are shown in 30-day intervals, from 01/01/2016 to 09/30/2021.

Supplemental Figure 2. Initiation of Oral Anticoagulation by Age and Race\Ethnicity.

**
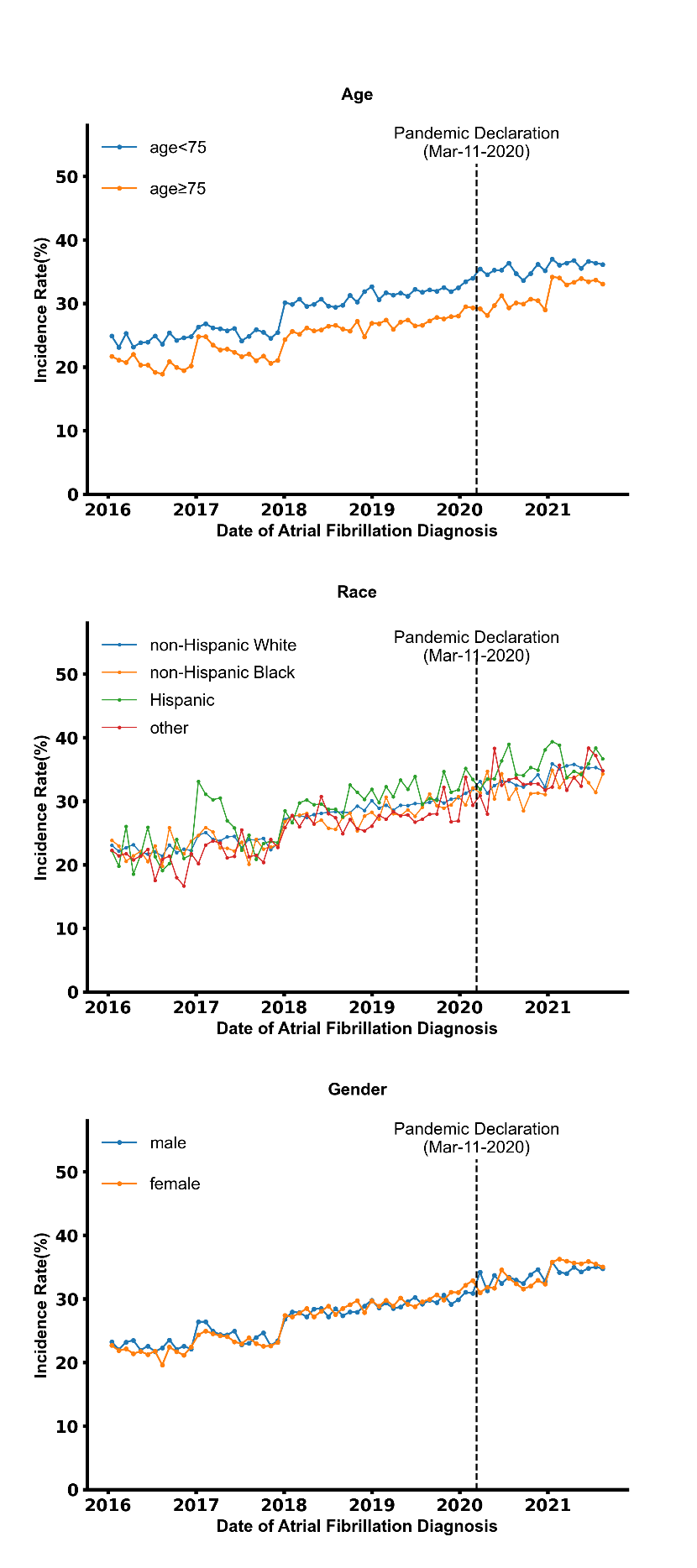
**

The figure shows trends in the initiation of oral anticoagulation within 30 days of atrial fibrillation diagnosis by age subgroup (upper panel), gender subgroup (middle panel) and race and ethnicity group (lower panel). Data are shown in 30-day intervals, from 01/01/2016 to 09/30/2021.
